# Supplementary material for: Altered microRNA Transcriptome in Cultured Human Liver Cells upon Infection with Ebola Virus
Source: Int J Mol Sci. 2021 Apr 6;22(7):3792. doi: 10.3390/ijms22073792 (PMC8038836; doi:10.3390/ijms22073792)
Supplement: Supplementary file 1 [file ijms-22-03792-s001.zip › Supplementary_File/D_ KEGG_Analysis_Results/16-30nt_pathway_Reston-24h-Huh7_vs_Control-24h-Huh7_down.mature_mirna_targets/hsa_GeneRatioDotPlot.pdf]

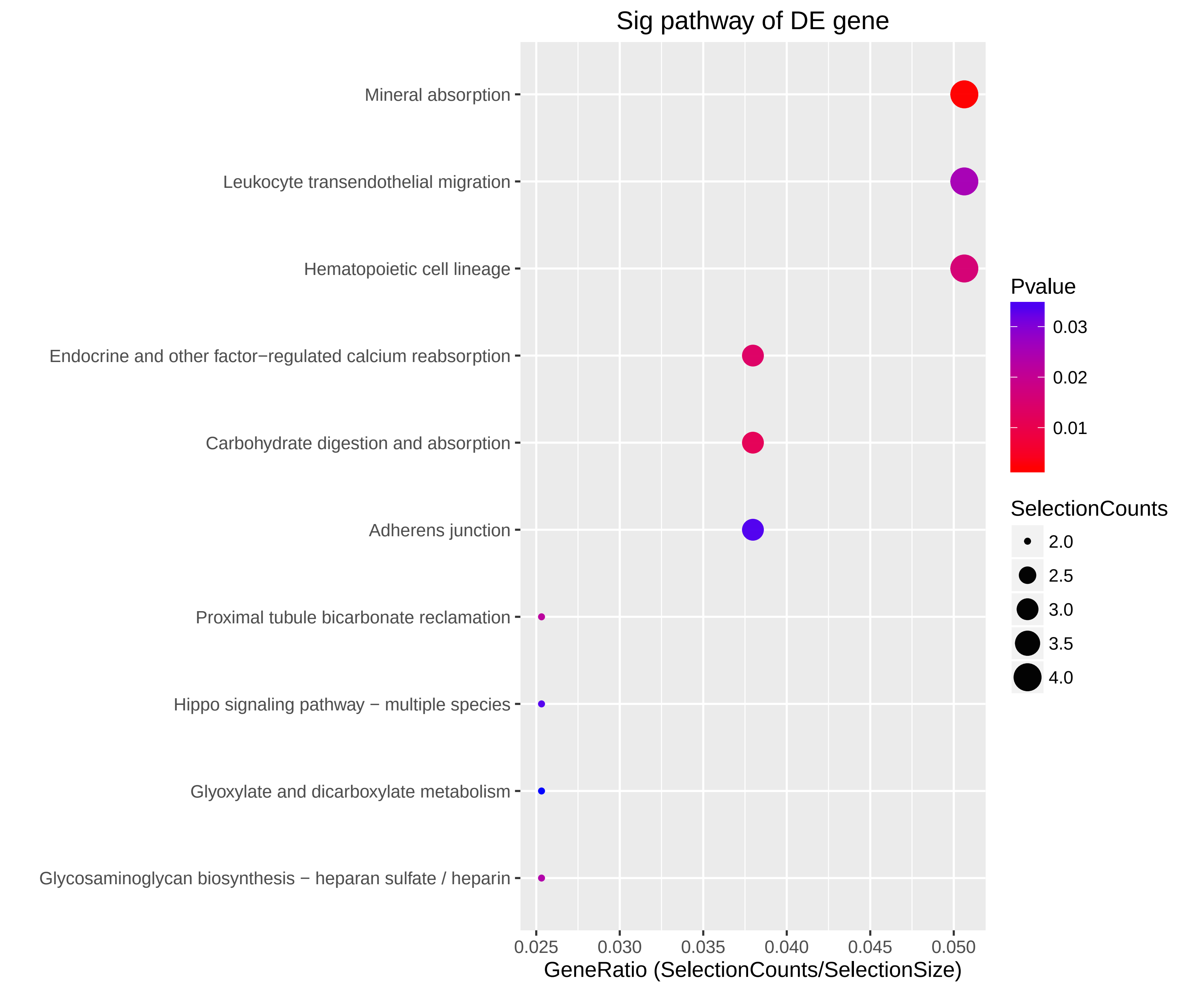
Sig pathway of DE gene
Mineral absorption
Leukocyte transendothelial migration
Hematopoietic cell lineage
Endocrine and other factor−regulated calcium reabsorption
Carbohydrate digestion and absorption
Adherens junction
Proximal tubule bicarbonate reclamation
Hippo signaling pathway − multiple species
Glyoxylate and dicarboxylate metabolism
Glycosaminoglycan biosynthesis − heparan sulfate / heparin
0.025
0.030
0.035
0.040
0.045
0.050
GeneRatio (SelectionCounts/SelectionSize)
Pvalue
0.03
0.02
0.01
SelectionCounts
2.0
2.5
3.0
3.5
4.0
